# CT-guided transforaminal epidural steroid injection for discogenic lumbar radiculopathy: influence of contrast dispersion and radiologist’s experience on clinical outcome

**DOI:** 10.1007/s00256-021-03881-x

**Published:** 2021-08-12

**Authors:** Christoph Germann, Dimitri N. Graf, Benjamin Fritz, Reto Sutter

**Affiliations:** Radiology, Balgrist University Hospital, University of Zurich, Forchstrasse 340, 8008 Zurich, Switzerland

**Keywords:** Radiculopathy, Injections, Steroids, Tomography, Treatment outcome

## Abstract

**Objective:**

To investigate the impact of contrast dispersion pattern/location during lumbar CT-guided transforaminal epidural steroid injection (TFESI) and experience of the performing radiologist on therapeutic outcome.

**Materials and methods:**

In this single-center retrospective cohort study, two observers analyzed contrast dispersion during CT-guided TFESI of 204 patients (age 61.1 ± 14 years) with discogenic unilateral single-level L4 or L5 radiculopathy. The contrast dispersion pattern was classified as “focal,” “linear,” or “tram-track”; the location was divided into “extraforaminal,” “foraminal,” or “recessal.” Pain was assessed before and 4 weeks after treatment using a numerical rating scale (0, no pain; 10, intolerable pain). Additionally, the patient global impression of change (PGIC) was assessed. The TFESI was performed by musculoskeletal radiologists (experience range: first year of musculoskeletal fellowship training to 19 years). Contrast pattern/location and radiologist’s experience were compared between “good responder” (≥ 50% pain reduction) and “poor responder” (< 50%). A *p*-value < 0.05 was considered to be statistically significant.

**Results:**

Overall, CT-guided TFESI resulted in a substantial pain reduction in 46.6% of patients with discogenic radiculopathy. The contrast dispersion pattern and location had no effect on pain relief (*p* = 0.75 and *p* = 0.09) and PGIC (*p* = 0.70 and *p* = 0.21) 4 weeks after TFESI. Additionally, the experience of the radiologist had no influence on pain reduction (*p* = 0.92) or PGIC (*p* = 0.75). Regarding pre-interventional imaging findings, both the location and grading of nerve compression had no effect on pain relief (*p* = 0.91 and *p* = 0.85) and PGIC (*p* = 0.18 and *p* = 0.31).

**Conclusion:**

Our results indicate that neither contrast agent dispersion/location nor the experience of the radiologist allows predicting the therapeutic outcome 4 weeks after the procedure.

## Introduction

Low back pain is a very common condition in the adult population, with a lifetime prevalence of 15–45%, and with an ample medical and socioeconomic burden [1]. Frequent causes of low back pain comprise disc herniations with compression of nerve roots, either at the level of the lateral recess, within the neuroforamen, or outside of the foramen (“extraforaminal”). By far, the two most commonly affected lumbar levels by disc herniation (> 95%) are L4–5 as well as L5–S1, and accordingly, disc herniation is the most common cause of L4 and L5 radiculopathy [2]. Many patients with lumbar radiculopathy respond favorably to imaging-guided therapeutic injections with steroids [3, 4]; therefore, these injections have steadily increased over the last decades [5–9]. Radicular pain is caused by mechanical compression of the nerve (e.g., due to disc herniation) resulting in local inflammatory processes [10]. Corticosteroid injections in radicular low back pain are used for their ability to inhibit this inflammatory cascade. Hence, the drugs are injected close to the pain source, i.e., the mechanically irritated, inflamed nerve root. Lumbar epidural steroid injections can be performed reliably, quickly, and safely with image guidance (fluoroscopy-guided or CT-guided) [11–17].

Several studies have investigated the correlation between the contrast dispersion pattern during fluoroscopy-guided transforaminal epidural steroid injection (TFESI) and therapeutic outcome after steroid injection [18–21]; however, study designs (patient inclusion, type and dosage of steroid agent, etc.) varied considerably, patient cohorts were rather small (*n* < 65), and results were contradictory. Although fluoroscopy was proven to yield less radiation exposure for patients during TFESI (although more radiation exposure for the radiologist) compared to CT, the radiation dose is still at a low level [14]. The widespread availability of CT scanners as well as the simplicity of CT-guided interventions with a steep learning curve makes CT the standard modality for image-guided lumbar steroid injections in many institutions, allowing the direct visualization of the needle, the nerve root, and other anatomical structures. Apart from exact placement of the needle tip adjacent to the affected nerve, there may be other factors during CT-guided TFESI, such as the pattern and location of the applied contrast agent and experience of the performing radiologist, which may influence the therapeutic efficacy. To our knowledge, the role of the contrast dispersion pattern and location in CT-guided lumbar TFESI regarding pain relief has not yet been investigated.

Thus, the aim of our study was to evaluate whether there is an association between contrast dispersion during CT-guided TFESI and therapeutic outcome (in patients with discogenic nerve compression, either in the lateral recess, in the foramen, or both), and secondarily if the radiologist’s experience influences the therapeutic efficacy.

## Materials and methods

### Study population

This prospective single-center cohort study with retrospective data analysis was approved by the local ethics committee. Potentially eligible subjects were identified by means of a database search in our hospital information system for patients with lumbar radiculopathy with subsequent transforaminal epidural steroid injections in our radiological department. Prior to the intervention, written informed consent was given regarding the procedure itself as well as the future use of data for research purposes.

Inclusion criteria comprised (1) TFESI for lumbar radiculopathy with or without low back pain (radiculopathy confirmed by clinical examination by board-certified orthopedic surgeons and rheumatologists); (2) discogenic nerve compression (either in the neuroforamen, at the lateral recess, or both) of the respective treated nerve root, confirmed by MRI within 3 months prior to the TFESI; (3) no prior lumbar steroid injection within 3 months; and (4) age ≥ 18 years. Patients were excluded if at least one of the following applied: (1) TFESI for lumbar radiculopathy other than L4 or L5, (2) bilateral or multilevel infiltration in the same session, (3) fluoroscopic guidance (instead of CT guidance), and (4) missing pain score data.

**Fig. 1:**
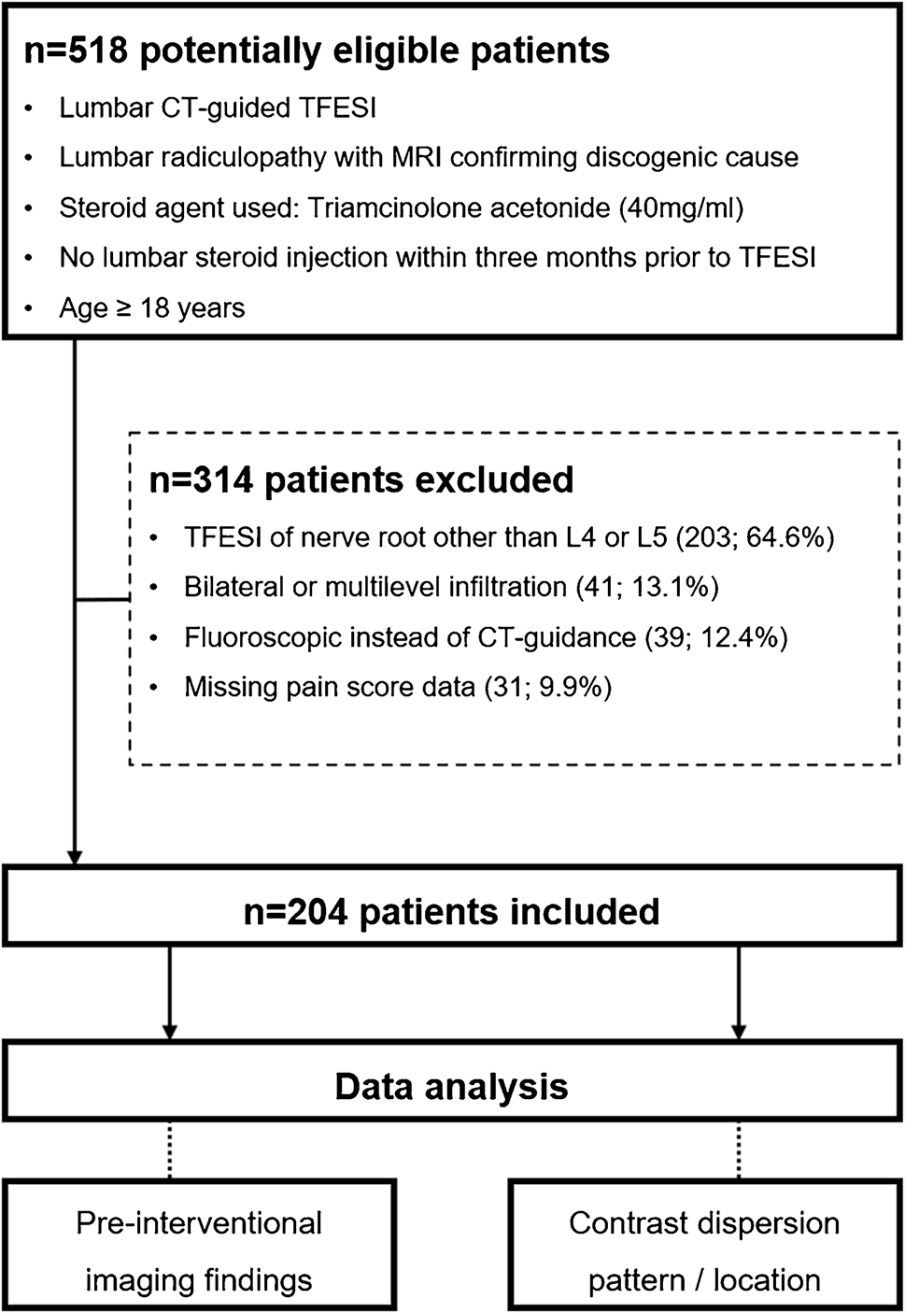
Flowchart of study design. TFESI, transforaminal epidural steroid injection

A detailed study flowchart for patient inclusion/exclusion is shown in Fig. 1.

### Outcome questionnaires

The outcome measurement has been performed prospectively as follows: each patient stated the current maximum pain level regarding the low back pain and/or radiating leg pain (whichever was more severe) immediately prior to the TFESI, using an 11-point numerical rating scale (NRS) with 0 meaning “no pain” and 10 “intolerable pain.” The pain score 4 weeks after the procedure was acquired by using a questionnaire that was given to the patient immediately after the TFESI, completed after 4 weeks and sent back to our department via prepaid post. Based on the therapeutic efficacy of the steroid injection, two groups were formed: (1) “good responder” with at least 50% reduction in NRS score 4 weeks after TFESI and (2) “poor responder” with below 50% reduction in NRS score 4 weeks after TFESI [17, 20]. In a subgroup analysis, each patient with a baseline NRS score (before CT-guided TFESI) of < 4—representing only mildly symptomatic patients—were excluded, as the likelihood to benefit from a steroid injection is inherently lower in these patients, potentially confounding the results. In addition to the NRS pain score, the patient global impression of change (PGIC, a seven-item scale), as a measure of the patient’s quality of life was assessed 4 weeks after the TFESI: participants were asked to rate the overall change in activity limitation, symptoms, emotions, and overall quality of life related to the low back pain and/or radiating leg pain after the steroid injection [14, 22, 23]. The possible answers included (1) “much worse,” (2) “worse,” (3) slightly worse,” (4) “no change,” (5) “slightly better,” (6) “better,” and (7) “much better.” The answers “much worse,” “worse,” and “slightly worse” were considered to represent relevant worsening, whereas “better” and “much better” indicated relevant improvement. The answers “no change” and “slightly better” represented no change [14, 22, 23]. Based on the PGIC score, two groups were formed: (1) patients with “relevant worsening” and (2) patients with “relevant improvement.”

### Lumbar injection procedure

All injections were performed as an outpatient procedure by 1 of 18 musculoskeletal radiologists in our institution (experience range: first year of musculoskeletal fellowship training to 19 years of specialized musculoskeletal practice). To ensure consistency and reproducibility, a standardized injection protocol was used: (1) initial lumbar low-dose CT in a prone position at the requested level, using a 64-detector row CT; (2) planning the access route for needle insertion and positioning by the radiologist using the initial CT; (3) aseptic preparation; (4) needle placement under CT guidance with the needle tip adjacent to the respective nerve root using a transforaminal approach; (5) ensuring correct needle tip position using iodized contrast agent, 1 mL iopamidol (Iopamiro 200, 200 mg/mL of iodine); (6) injection of 40 mg (1 mL) of triamcinolone acetonide and 1 mL of 0.2% lidocaine. Despite an FDA warning in 2014 regarding the safety of epidural use of particulate steroids, we mainly use triamcinolone in our institution for lower lumbar TFESI based on (a) large cohort studies which show only minor adverse events after lumbar epidural injection similar to non-particulate steroids [24, 25] and (b) the higher efficacy of particulate steroids (e.g., triamcinolone) compared to that of non-particulate steroids (e.g., dexamethasone) [3]. After the procedure, the CT images were archived in the picture archiving and communication system (PACS).

The effective dose (in mSv) for each CT-TFESI was calculated by multiplying the dose-length product (provided by the patient protocol of the CT scanner) by the conversion factor of 0.0127 mSv/mGy/cm [26].

### Contrast dispersion assessment on CT

**Fig. 2:**
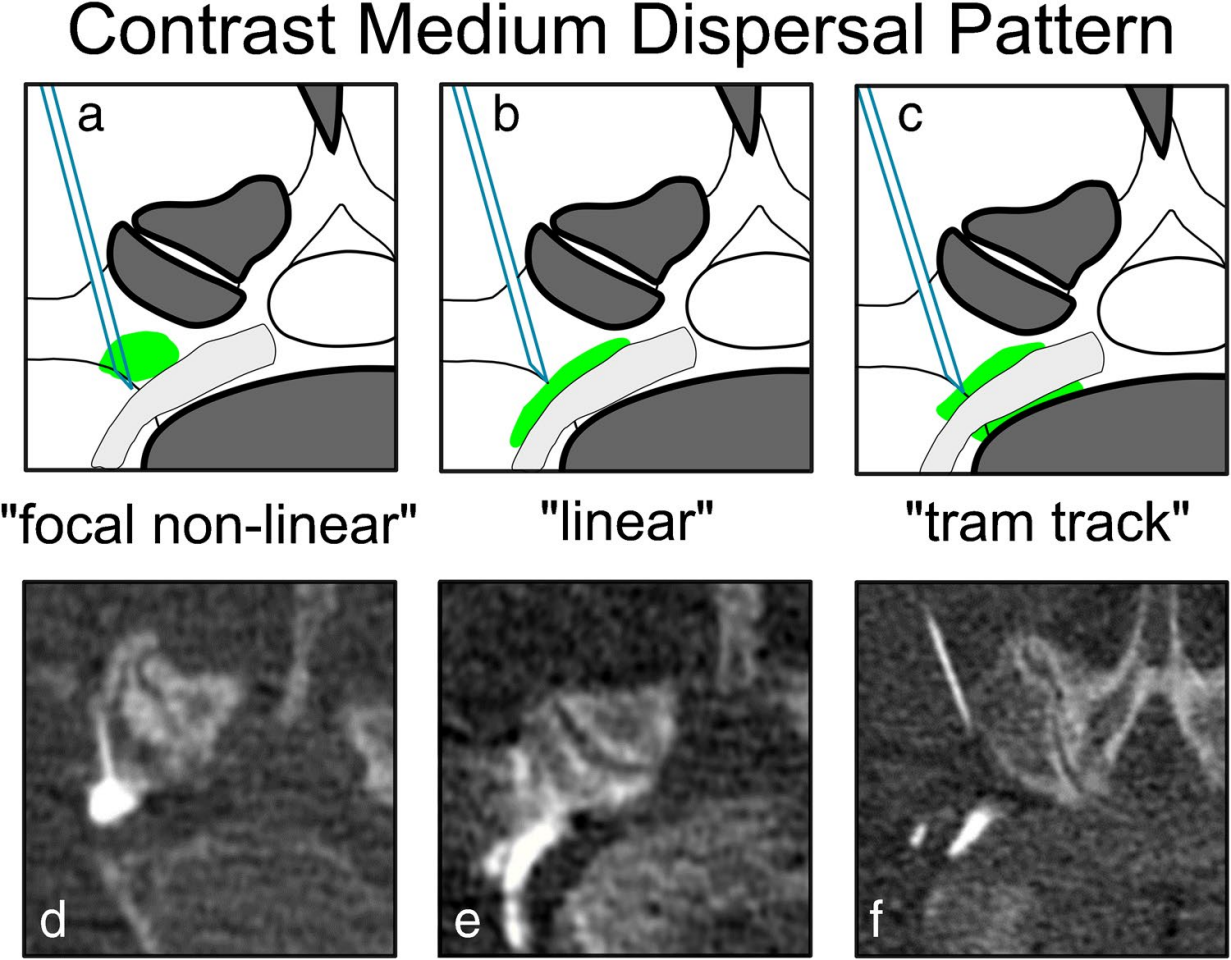
Schematic drawing (**a**–**c**) of the three possible contrast medium dispersal patterns (green color in schematic drawings) occurring during TFESI with corresponding axial CT images (**d**–**f**.) **a** and **d** show “focal non-linear” contrast agent distribution with contact to the spinal nerve, **b** and **e** illustrate “linear” contrast agent spreading along the spinal nerve, and **c** and **f** depict the “tram-track” type of contrast medium dispersion along the spinal nerve. TFESI, transforaminal epidural steroid injection

**Fig. 3:**
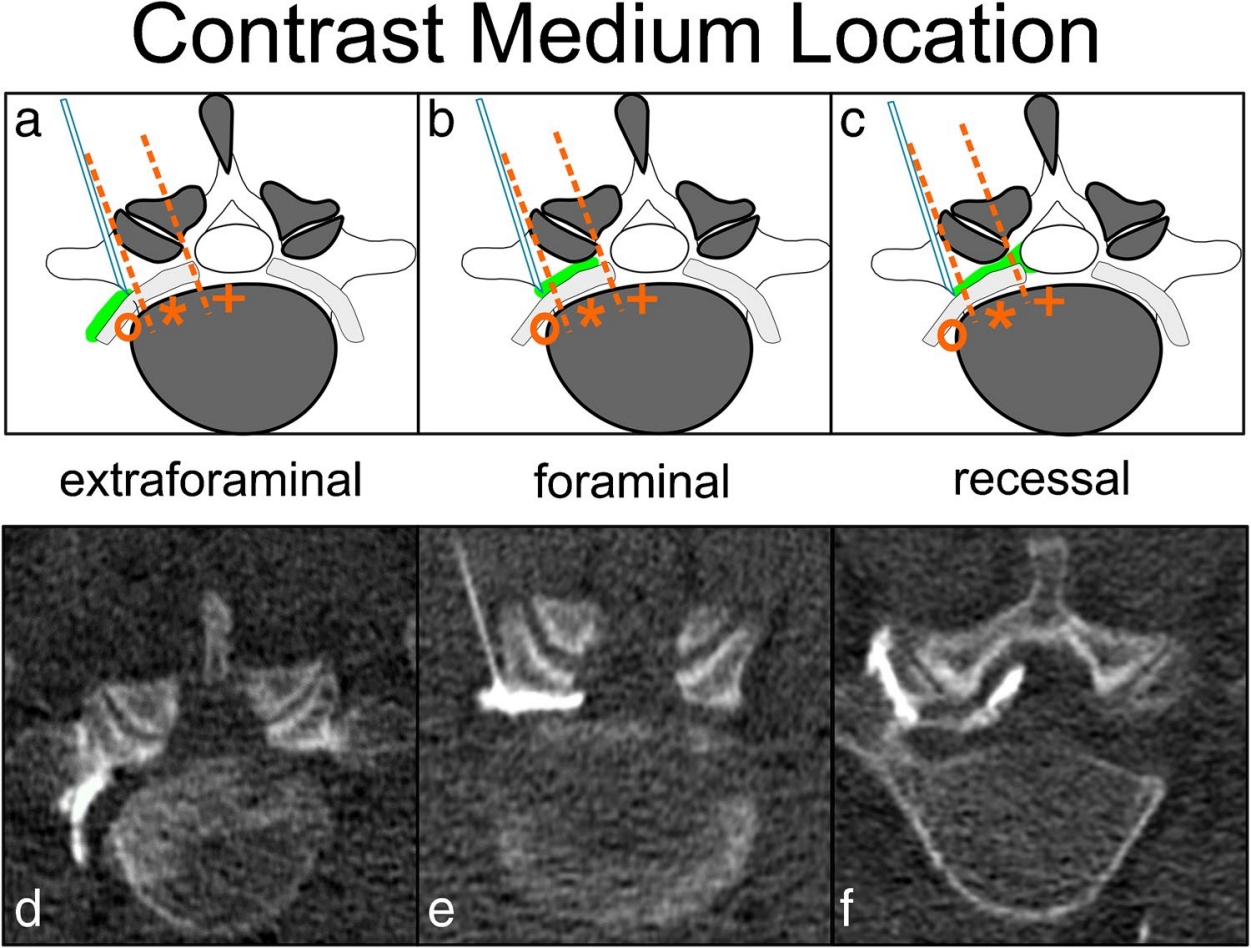
Schematic drawing (**a**–**c)** of the three different possible locations of the contrast medium (green color in schematic drawing) during TFESI with corresponding axial CT images (**d–f**). Orange dashed lines illustrate the distinction between the locations “extraforaminal” (circle), “foraminal” (asterisk), and “recessal” (cross). **a** and **d** represent the “extraforaminal” location of contrast, **b** and **e** depict mainly “foraminal” contrast, and **c** and **f** show the contrast reaching the “recessal” compartment. TFESI, transforaminal epidural steroid injection

Two fellowship-trained musculoskeletal radiologists (with 7 and 8 years of experience, respectively) analyzed the contrast dispersion on the CT image data sets. Evaluations were performed in an independent and randomized fashion on anonymized data sets using state-of-the-art PACS workstations. Both radiologists were blinded to all clinical data, including the pre- and post-procedure pain scores/PGIC scores. The contrast dispersion pattern was classified as “focal non-linear” when a focal nodular accumulation was seen, as “linear” when the dispersion was threadlike along one side of the spinal nerve, and as “tram-track” when the contrast agent was found both anterior and posterior to the spinal nerve (Fig. 2). The contrast agent location was divided into either “extraforaminal” when the contrast agent was located exclusively outside the neuroforamen along the nerve; “foraminal” when the contrast agent was located mainly along the nerve within the neuroforamen, without reaching the recess of the nerve root; or “recessal” when the contrast agent reached the spinal lateral recess of the nerve root (Fig. 3).

### Pre-procedural lumbar imaging findings

Two fellowship-trained musculoskeletal radiologists (with 7 and 8 years of experience, respectively) performed the image analysis independently on anonymized data sets, blinded to all clinical data and to the CT-guided TFESI procedure, evaluating the following parameters: (a) presence of concomitant osseous nerve root compromise (e.g., due to spondylosis, osteoarthrosis); (b) location of discogenic nerve root compromise (L4 or L5; lateral recess, neuroforamen, or both); and (c) grading of nerve root compression: grade 0 = no compromise/stenosis, grade 1 = contact of disc material with nerve root/mild stenosis, grade 2 = deviation of nerve root/moderate stenosis, and grade 3 = compression of nerve root/severe stenosis [27, 28].

### Clarification of nomenclature regarding nerve root levels

In case of L4 radiculopathy, the discogenic nerve compression was present either within the neuroforamen, where the L4 nerve roots exit (level L4–5), at the lateral recess of the L4 nerve root (level L3–4), or both. Accordingly, L5 radiculopathy was caused by either a foraminal discogenic compression of the exiting L5 nerve root (level L5–S1), compression at the lateral recess of the L5 nerve root (level L4–5), or both. CT-guided steroid injection was performed exclusively using a transforaminal approach; hence, L4 radiculopathy was treated at level L4–5; and L5 radiculopathy was treated at level L5–S1, irrespective of the location of discogenic nerve compromise (neuroforamen, lateral recess, or both).

### Statistical analysis

Statistical analysis was performed using SPSS (v25, IBM Corp., Somers, NY). General descriptive statistics were applied. If not stated otherwise, categorical/ordinal data are presented as proportions/percentages and continuous data as means with standard deviation. A Kolmogorov–Smirnov test was applied to test for normal distribution. The Mann–Whitney *U* test was used to compare continuous, ordinal, and categorical (non-binary) variables between groups, and chi-square test was used to compare binary variables. Pearson’s correlation was used to test for an association between the radiologist’s experience and pain relief as well as radiation exposure. Multivariate regression analysis was applied to test for a significant correlation between all available variables (demographics, radiological pre-procedural MRI findings, and intraprocedural contrast dispersion-based findings) and patient outcome (pain score and/or PGIC). A *p*-value < 0.05 was considered to represent statistical significance.

Inter-reader agreement was assessed by calculating Cohen’s kappa and interpreted according to Landis and Koch as either “slight” (0–0.20), “fair” (0.21–0.40), “moderate” (0.41–0.60), “substantial” (0.61–0.80), or “almost perfect” agreement (0.81–1.00) [29].

## Results

### Inter-reader agreement

The agreement between both radiologists was substantial for the location of nerve compression (*ĸ* = 0.79) and almost perfect for the grading of nerve stenosis (*ĸ* = 0.85), presence of concomitant osseous stenosis (*ĸ* = 0.90), contrast dispersion pattern (*ĸ* = 0.91), and contrast agent location (*ĸ* = 0.96).

### Study population

Two hundred four patients with CT-guided TFESI for unilateral L4 or L5 radiculopathy were enrolled (Fig. 1). The mean age of all participants was 61.1 ± 14.0 years, 101 of 204 (49.5%) were male, and 103 of 204 (50.5%) were female. One hundred four of 204 (51.0%) patients had left-sided TFESI, 100 of 204 (49.0%) had right-sided TFESI, 53 of 204 TFESI (26.0%) were at the L4 level, and 151 of 204 (74.0%) at the L5 level.

The baseline NRS score was 5.9 ± 2.3; the NRS score 4 weeks after TFESI was 3.7 ± 2.6.

Overall, 95 of 204 (46.6%) patients were “good responder” (≥ 50% pain reduction) and 109 of 204 (53.4%) “poor responder” (< 50% pain reduction) 4 weeks after TFESI (Table 1). There was a substantial reduction of pain levels for “good responder” with NRS scores of 6.5 ± 2.1 at baseline and NRS scores of 1.8 ± 1.3 after 4 weeks, while the “poor responder” reported pain levels of 5.4 ± 2.4 at baseline, and 5.4 ± 2.3 after 4 weeks.

**Table 1:** Demographics

Variable	Good responder *N* = 95	Poor responder *N* = 109	*p*-value
Age, *years*	60.1 ± 13.9	61.3 ± 14.1	0.98
BMI, *kg/m*^*2*^	26.6 ± 4.1	26.7 ± 4.7	0.74
Sex, *n (*%*)*			
Male	49 (51.6)	52 (47.7)	0.58
Female	46 (48.4)	57 (52.3)	
*Level*, *n (*%*)*			
L4	25 (26.3)	28 (25.7)	0.92
L5	70 (73.7)	81 (74.3)	
Side, *n (*%*)*			
Left	41 (43.2)	63 (57.8)	0.04*
Right	54 (56.8)	46 (42.2)	
*Prior steroid injection*, *n *(%)			
Yes	23 (24.2)	27 (24.8)	0.93
No	72 (75.8)	82 (75.2)	
*Pain assessment*			
NRS baseline	6.5 ± 2.1	5.4 ± 2.4	0.002*
NRS after 4 weeks	1.8 ± 1.3	5.4 ± 2.3	< 0.001*

Comparison of variables “age,” “sex,” “level of TFESI,” “side of TFESI,” “prior steroid injection” in the lumbar spine, and “pain assessment” between groups “good responder” and “poor responder” is shown. Numerical data are presented as mean ± standard deviation, and categorical data as numbers (percentages)

*TFESI* transforaminal epidural steroid injection, *NRS* numerical rating scale

*Denotes statistical significance (*p* < 0.05)

Based on the PGIC score 4 weeks after TFESI, 23 of 204 (11.3%) patients had “relevant improvement,” as opposed to 135 of 204 (66.2%) with “relevant worsening”; the remaining 46 of 204 (22.5%) were considered to have “no change” after TFESI.

### Pre-interventional imaging findings

**Fig. 4:**
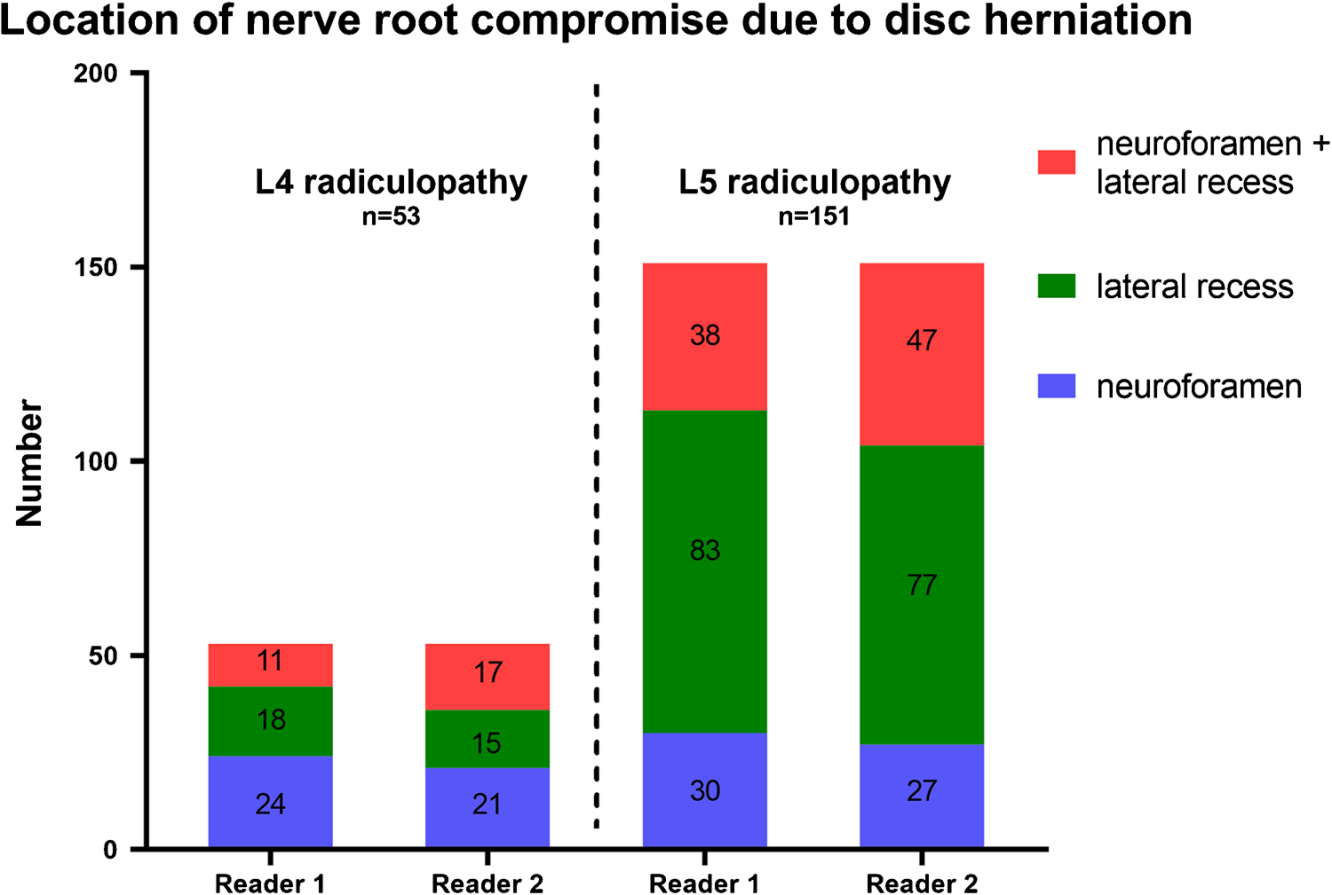
Bar chart of *n* = 204 CT-TFESI procedures performed for L4 radiculopathy (*n* = 53) and L5 radiculopathy (*n* = 151) caused by disc herniation. The location of discogenic nerve root compromise for all 204 patients is shown for reader 1 and reader 2: numbers represent cases treated due to discogenic radiculopathy with nerve root compromise either both in the neuroforamen and lateral recess (red), only in the lateral recess (green), or only in the neuroforamen (blue). TFESI, transforaminal epidural steroid injection

All included patients had pre-interventional MR imaging showing a discogenic compromise of the respective nerve root, consistent with the level and side of radiculopathy treated. Additionally, 45 of 204 (22.1%) patients (44 of 204 (21.6%) for reader 2) were rated to have a concomitant osseous component of nerve compression. The nerve compression was located in the neuroforamen in 54 of 204 (26.5%) patients (48 of 204 (23.5%) for reader 2), in the lateral recess in 101 of 204 (49.5%) patients (92 of 204 (45.1%) for reader 2), and both foraminal and in the lateral recess in 49 of 204 (24.0%) patients (64 of 204 (31.4%) for reader 2). Figure 4 illustrates the location of nerve compromise in all cases treated for L4 radiculopathy and L5 radiculopathy. Nerve root compression was graded as “grade 1” in 19 of 204 (9.3%) patients (14 of 204 (6.9%) for reader 2), “grade 2” in 73 of 204 (35.8%) patients (84 of 204 (41.2%) for reader 2), and “grade 3” in 112 of 204 (54.9%) patients (106 of 204 (52.0%) for reader 2).

### Findings during CT-guided TFESI

The effective dose for the TFESI procedures was 0.43 ± 0.20 mSv. No correlation was found between the experience of the performing radiologist and radiation exposure for the patient during CT-TFESI (*r* = 0.07; *p* = 0.44).

For reader 1/reader 2, the contrast dispersion pattern was “focal non-linear” in 55 of 204/48 of 204 cases (27.0%/23.5%), “linear” in 133 of 204/141 of 204 cases (65.2%/69.1%), and “tram-track”-like in 16 of 204/15 of 204 cases (7.8%/7.4%), respectively. For reader 1/reader 2, the contrast agent location during TFESI was “extraforaminal” in 137 of 204/136 of 204 cases (67.2%/66.7%), “foraminal” in 34 of 204/36 of 204 cases (16.2%/17.6%), and “recessal” in 33 of 204/32 of 204 cases (16.2%/15.7%), respectively.

### Influence of demographic variables, imaging findings, and radiologist experience on pain relief

Regarding patient demographics, the side of radiculopathy was significantly different between “good responder” (left 41, right 54) and “poor responder” (left 63, right 46; *p* = 0.04). The baseline NRS was slightly but significantly lower in the group “poor responder” (5.4 ± 2.4) compared to “good responder” (6.5 ± 2.1; *p* = 0.002) (Table 1).

No pre-interventional imaging finding was associated with a significantly better therapeutic outcome 4 weeks after CT-guided TFESI (Table 2): the presence of concomitant osseous stenosis (*p* = 0.30/*p* = 0.23 for reader 1/2), the location of nerve compression (*p* = 0.61/*p* = 0.75 for reader 1/2), and the grade of nerve compression (*p* = 0.95/*p* = 0.92 for reader 1/2) were not significantly different between “good responder” and “poor responder.”

**Table 2:** Imaging findings and TFESI-related findings

Variable	Good responder *N* = 95	Poor responder *N* = 109	*p*-value
*Concomitant osseous nerve compression*, *n (*%*)*			
*Reader 1/reader 2*			0.30/0.23
Yes	24 (25.3)/24 (25.3)	21 (19.3)/20 (18.3)	
No	71 (74.7)/71 (74.7)	88 (80.7)/89 (81.7)	
*Location nerve compression*, *n (*%*)*			
*Reader 1/reader 2*			0.61/0.75
Foramen	25 (26.3)/21 (22.1)	29 (26.6)/27 (24.8)	
Recess	50 (52.6)/47 (49.5)	51 (46.8)/45 (41.3)	
Both	20 (21.1)/27 (28.4)	29 (26.6)/37 (33.9)	
*Stenosis* (grade), *n (*%*)*			
*Reader 1/reader 2*			0.95/0.92
Grade 1	9 (9.5)/7 (7.4)	10 (9.2)/7 (6.4)	
Grade 2	34 (35.8)/38 (40.0)	39 (35.8)/46 (42.2)	
Grade 3	52 (54.7)/50 (52.6)	60 (55.0)/56 (51.4)	
*Contrast dispersion pattern*, *n *(%)			
*Reader 1/reader 2*			0.97/0.58
Focal non-linear	27 (28.4)/26 (27.3)	28 (25.7)/22 (20.2)	
Linear	59 (62.1)/60 (63.2)	74 (67.9)/81 (74.3)	
Tram-track	9 (9.5)/9 (9.5)	7 (6.4)/6 (5.5)	
*Contrast location*, *n *(%)			
*Reader 1/reader 2*			0.19/0.11
Extraforaminal	67 (70.5)/68 (71.6)	70 (64.2)/68 (62.4)	
Foraminal	18 (18.9)/17 (17.9)	16 (14.7)/19 (17.4)	
Recessal	10 (10.5)/10 (10.5)	23 (21.1)/22 (20.2)	
*Experience level*, *n (*%)			
Fellow	26 (27.4)	30 (27.5)	0.98
Consultant	69 (72.6)	79 (72.5)	

The variables “concomitant osseous nerve compression,” “location of nerve compression,” “stenosis grade,” “contrast dispersion pattern,” “contrast medium location,” and “experience of radiologist” are compared between the two groups “good responder” and “poor responder.” Data are presented as numbers (percentages)

*TFESI* transforaminal epidural steroid injection

The contrast dispersion pattern during TFESI had no predictive value on pain relief (Table 2): the “good responder”/ “poor responder” subgroups showed 28.4%/25.7% “focal non-linear,” 62.1%/67.9% “linear,” and 9.5%/6.4% “tram-track” patterns (*p* = 0.97; data for reader 1; data for reader 2 are given in Table 2).

The contrast agent location during TFESI was not significantly different between both groups: “good responder”/ “poor responder” showed 70.5%/64.2% “extraforaminal,” 18.9%/14.7% “foraminal,” and 10.5%/21.1% “recessal” contrast locations (*p* = 0.19; data for reader 1; data for reader 2 are given in Table 2).

Fifty-six of the 204 TFESI (27.5%) have been performed by radiologists within their first year of musculoskeletal fellowship training, and 148 of 204 (72.5%) by consultant-level radiologists (> 1-year of musculoskeletal subspecialization), respectively. The mean experience level was 3.8 ± 3.8 years of musculoskeletal practice (range 1st to 19th year). The experience level showed no correlation with the therapeutic outcome after 4 weeks (*p* = 0.98; Table 2).

A multivariate regression analysis found no significant correlation for any abovementioned demographic or radiological findings and pain relief after CT-guided TFESI (Table 3).

**Table 3:** Multivariate regression analysis: influence on pain relief

Variable	*Regression coefficient beta*	*Standard error*	*p-value*
Age	0.003 (0.004)	0.012 (0.012)	*0.83 (0.73)*
BMI	0.016 (0.016)	0.035 (0.035)	*0.66 (0.66)*
Sex	0.056 (0.061)	0.31 (0.31)	*0.86 (0.85)*
Level	0.011 (0.055)	0.36 (0.36)	*0.98 (0.88)*
Side	0.46 (0.45)	0.31 (0.31)	*0.14 (0.15)*
Prior steroid injection	0.066 (0.072)	0.36 (0.36)	*0.85 (0.84)*
Concomitant osseous stenosis	0.14 (0.088)	0.40 (0.048)	*0.73 (0.83)*
Location nerve compression	0.027 (0.027)	0.23 (0.22)	*0.91 (0.90)*
Stenosis grade	0.043 (0.025)	0.23 (0.25)	*0.85 (0.92)*
Contrast dispersion pattern	0.094 (0.072)	0.30 (0.31)	*0.75 (0.82)*
Contrast location	0.40 (0.42)	0.23 (0.23)	*0.09 (0.07)*
Experience of radiologist	0.032 (0.033)	0.36 (0.37)	*0.92 (0.89)*
*R-squared*	0.053 (0.058)		

Data are presented for reader 1 (reader 2). Effect of “age,” “BMI,” “sex,” “level of radiculopathy/treatment,” “affected side,” “prior lumbar steroid injection,” “concomitant osseous stenosis,” “location of nerve compression,” “stenosis grade,” “contrast dispersion pattern,” “contrast location,” and “experience of radiologist” on pain relief 4 weeks after CT-guided TFESI

*TFESI* transforaminal epidural steroid injection

### *Subgroup analysis (baseline NRS score* < *4)*

In a subgroup analysis, all patients with a baseline NRS score of < 4 were excluded, as these patients were only mildly symptomatic with presumably reduced likelihood of being classified as “good responders” (≥ 50% pain reduction) after the CT-guided TFESI, thereby potentially confounding results: 164 of 204 patients met the additional inclusion criterion.

Neither the presence of concomitant osseous stenosis (*p* = 0.17), location of nerve compression (*p* = 0.66), stenosis grade (*p* = 0.67), contrast dispersion pattern during CT-TFESI (*p* = 0.59), contrast location during CT-TFESI (*p* = 0.24), nor experience level of the performing radiologist (*p* = 0.80) (data for reader 1) was associated with a significantly better pain relief 4 weeks after TFESI (Table 4).

**Table 4:** Imaging findings and TFESI-related findings in subgroup (baseline NRS score ≥ 4)

Variable	Good responder *N* = 85	Poor responder *N* = 79	*p*-value
*Concomitant osseous nerve compression*, *n *(%)			
*Reader 1/reader 2*			0.17/0.15
Yes	24 (28.2)/23 (27.1)	15 (19.0)/14 (17.7)	
No	61 (71.8)/62 (72.9)	64 (81.0)/65 (82.3)	
*Location nerve compression*, *n *(%)			
*Reader 1/reader 2*			0.66/0.78
Foramen	22 (25.9)/19 (22.4)	19 (24.1)/18 (22.8)	
Recess	45 (52.9)/42 (49.4)	41 (51.9)/36 (45.6)	
Both	18 (21.2)/24 (28.2)	19 (24.1)/25 (31.6)	
*Stenosis* (*grade*), *n (*%*)*			
*Reader 1/reader 2*			0.67/0.39
Grade 1	9 (10.6)/7 (8.2)	9 (11.4)/7 (8.9)	
Grade 2	30 (35.3)/33 (38.8)	30 (38.0)/36 (45.6)	
Grade 3	46 (54.1)/45 (52.9)	40 (50.6)/36 (45.6)	
*Contrast dispersion pattern*, *n *(%)			
*Reader 1/reader 2*			0.59/0.32
Focal non-linear	25 (29.4)/25 (29.4)	17 (21.5)/14 (17.7)	
Linear	51 (60.0)/51 (60.0)	57 (72.2)/60 (75.9)	
Tram-track	9 (10.6)/9 (10.6)	5 (6.3)/5 (6.3)	
*Contrast location*, *n *(%)			
*Reader 1/reader 2*			0.24/0.13
Extraforaminal	59 (69.4)/60 (70.6)	50 (63.3)/48 (60.8)	
Foraminal	17 (20.0)/16 (18.8)	12 (15.2)/15 (19.0)	
Recessal	9 (10.6)/9 (10.6)	17 (21.5)/16 (20.3)	
*Experience level*, *n *(%)			
Fellow	23 (27.1)	20 (25.3)	0.80
Consultant	62 (72.9)	59 (74.7)	

In contrast to Table 2, data illustrated in this table represent a subgroup of the cohort after excluding patients with a baseline NRS score of < 4. The variables “concomitant osseous nerve compression,” “location of nerve compression,” “stenosis grade,” “contrast dispersion pattern,” “contrast medium location,” and “experience of radiologist” are compared between the two groups “good responder” and “poor responder.” Data are presented as numbers (percentages)

*TFESI* transforaminal epidural steroid injection

### Influence of demographic variables and imaging findings on PGIC

Using a multivariate regression analysis, no significant association was found between demographic variables or radiological findings and patient global impression of change (PGIC): age (*p* = 0.74), BMI (*p* = 0.49), sex (*p* = 0.37), level of radiculopathy (*p* = 0.95), side (*p* = 0.83), prior lumbar steroid injection (*p* = 0.92), concomitant osseous stenosis (*p* = 0.42 for reader 1; *p* = 0.31 for reader 2), location of nerve compression (*p* = 0.18), stenosis grade (*p* = 0.31), contrast dispersion pattern (*p* = 0.70), contrast location (*p* = 0.21), and experience of radiologist (*p* = 0.75) (data for reader 1) showed no influence on PGIC (Table 5).

**Table 5:** Multivariate regression analysis: influence on patient global impression of change (PGIC)

Variable	Regression coefficient beta	Standard error	*p*-value
Age	0.003 (0.001)	0.010 (0.010)	0.74 (0.95)
BMI	0.020 (0.020)	0.029 (0.029)	0.49 (0.49)
Sex	0.23 (0.24)	0.26 (0.25)	0.37 (0.36)
Level	0.018 (0.026)	0.29 (0.29)	0.95 (0.93)
Side	0.055 (0.067)	0.25 (0.25)	0.83 (0.79)
Prior steroid injection	0.028 (0.054)	0.29 (0.29)	0.92 (0.85)
Concomitant osseous stenosis	0.26 (0.33)	0.32 (0.32)	0.42 (0.31)
Location nerve compression	0.25 (0.24)	0.19 (0.18)	0.18 (0.18)
Stenosis grade	0.20 (0.29)	0.19 (0.20)	0.31 (0.15)
Contrast dispersion pattern	0.093 (0.28)	0.25 (0.25)	0.70 (0.26)
Contrast location	0.23 (0.20)	0.18 (0.18)	0.21 (0.27)
Experience of radiologist	0.093 (0.077)	0.29 (0.29)	0.75 (0.79)
*R-squared*	0.040 (0.052)		

Data are presented for reader 1 (reader 2). Effect of “age,” “BMI,” “sex,” “level of radiculopathy/treatment,” “affected side,” “prior lumbar steroid injection,” “concomitant osseous stenosis,” “location of nerve compression,” “stenosis grade,” “contrast dispersion pattern,” “contrast location,” and “experience of radiologist” on PGIC 4 weeks after CT-guided TFESI

*PGIC* patient global impression of change, *TFESI* transforaminal epidural steroid injection

## Discussion

Lumbar transforaminal epidural steroid injection (TFESI) is a frequently performed and effective procedure in patients with discogenic lumbar radiculopathy. So far, only a few fluoroscopy-based studies with partly conflicting results regarding the influence of contrast agent dispersion/location during lumbar TFESI on the therapeutic outcome are available, but no analysis of contrast agent dispersion after lumbar CT-guided TFESI has been reported in the literature. Based on the presumption that steroid agents need to be in as much contact with the locally inflamed nerve root (due to discogenic compression), to enhance patient outcome after TFESI, the contrast dispersion pattern and location during TFESI might play an important role and predict different outcomes. Hence, in this study we examined the influence of different contrast dispersion patterns/locations during CT-guided lumbar TFESI on pain relief and patient global impression of change 4 weeks after the procedure. Secondarily, we investigated the impact of the performing radiologist’s experience in CT-guided TFESI on therapeutic outcome.

Overall, almost half of patients (46.6%) showed a significant improvement of pain score levels (at least 50% NRS reduction) 4 weeks after TFESI, which is consistent with findings in studies with similar study populations [3, 12, 30, 31]. However, only a minority of patients (11.3%) had “relevant improvement” based on their global impression of change (PGIC)—a therapeutic outcome score which reflects activity limitation, symptoms, emotions, and overall quality of life related to their low back pain and/or radiating leg pain. Hence, the TFESI seems to reduce the pain level more than it affects the overall quality of life/activity limitation. As discogenic nerve compression results in local inflammation of the compressed nerve root, one might presume that in order to enhance patient outcome during TFESI, the applied steroid agent needs to be spread as widely along the surface of the targeted nerve root as possible. Based on this presumption, one might reposition the needle tip based on unfavorable contrast dispersion during TFESI (i.e., focal non-linear pattern), although the needle tip is already adjacent to the nerve root. However, our results indicate that neither the contrast agent dispersion pattern nor contrast agent location during lumbar CT-guided TFESI can predict the therapeutic outcome (pain relief and overall impression of change) 4 weeks after the procedure. Consequently, our study suggests that—given a correct placement of the needle tip adjacent to the targeted nerve root—the needle does not need to be repositioned based on the contrast dispersion pattern/location during CT-guided lumbar TFESI in order to improve clinical outcome. Furthermore, all available demographic variables, such as age, BMI, sex, level of radiculopathy, and prior lumbar steroid injections, had no influence on therapeutic outcome.

Several studies with different study designs (e.g., type and dosage of steroid used, inclusion criteria) have investigated the influence of fluoroscopic factors during TFESI on pain relief, revealing conflicting results [18–21, 32]. One retrospective fluoroscopy-guided study which included 38 participants detected that a focal non-linear “paraneural” contrast agent dispersion pattern was associated with significantly better pain relief as opposed to a linear “paraneural” pattern [21]; however, (1) pre-interventional MRI to confirm discogenic radiculopathy was not available for each included patient, (2) a different drug was used (non-particulate steroid: methylprednisolone 40 mg), and (3) post-interventional pain score assessment was performed after 2 and 8 weeks. In contradiction, another retrospective fluoroscopy-guided study with 51 participants with lumbar radiculopathy using 20 mg triamcinolone acetonide showed that neither the contrast agent dispersion pattern nor contrast location is an independent predictor of the therapeutic outcome after TFESI for discogenic lumbar radiculopathy [20], which is consistent with our results. Another retrospective fluoroscopy-guided study (*n* = 64) disclosed similar results with no significant correlation between the contrast dispersion pattern and pain relief 2 weeks and 3 months after TFESI [32]; however, a different non-particulate steroid agent (betamethasone acetate, 6 mg) was used and no inclusion criteria regarding the cause for the lumbar radiculopathy (e.g., discogenic) were given, therefore potentially representing a heterogeneous patient cohort, requiring careful interpretation of these findings.

Regarding MR imaging findings in patients with discogenic lumbar radiculopathy, we found no significant correlation with post-therapeutic pain relief after 4 weeks for location or grade of nerve compression. This confirms the results of a recent study comparing transforaminal with interlaminar epidural steroid injections in 198 patients and another study with 209 patients receiving triamcinolone acetate for lumbar radiculopathy, both showing no association between MR findings and treatment outcome [9, 17]. Similarly, regarding cervical transforaminal epidural steroid injections, Lee and colleagues found no association between pre-procedural MRI findings (i.e., disc herniation or spondylosis with foraminal stenosis) and pain relief after 4 weeks [33]. Spondylosis/osteoarthrosis as a chronic cause of nerve compression (besides disc herniation) may arguably influence therapeutic outcome after steroid injections for lumbar radiculopathy. In our cohort, one in five patients had concomitant osseous stenosis in addition to discogenic nerve compromise. However, we found no association between the presence of concomitant osseous nerve compression and therapeutic outcome 4 weeks after TFESI.

Interestingly, our data suggest that the experience of the musculoskeletal radiologist performing the CT-guided TFESI procedure has no influence on pain reduction after 4 weeks, which may be due to the use of a standardized protocol for administering the CT-guided TFESI. Also, these findings might differ for other populations of radiologists, e.g., general non-subspecialized radiologists.

Our study has limitations. First, 18 different radiologists performed the TFESI in our cohort, potentially causing a certain variability in the procedure itself; however, each radiologist received musculoskeletal fellowship training in the same institution using a strict standardized interventional protocol, and a correct location of the needle tip adjacent to the respective nerve root was confirmed with CT in all patients. Second, this study included only single-level unilateral L4 or L5 radiculopathy and no patients with midline pain without radiculopathy which may limit generalizability of the findings to other lumbar levels, patients with bilateral radiculopathy, and/or patients with midline pain without radiculopathy. Third, our cohort comprised only patients with CT-guided TFESI, but it might be interesting to compare CT guidance with fluoroscopy guidance regarding patient outcome: the cross-sectional benefit of CT may facilitate the reliable positioning of the needle tip adjacent to the targeted nerve root; however, based on our findings, which imaging guidance method (fluoroscopy or CT) is used seems not to matter if the needle tip is placed directly at the nerve root. Nonetheless, this might be interesting to investigate systematically in future studies. Despite these limitations, with a cohort size of *n* = 204, which is considerably larger than those of previous studies, the standardized TFESI procedure with use of identical amounts of contrast agent and injected drugs as well as image evaluation by two board-certified musculoskeletal radiologists establishes a firm basis for the validity of our findings.

In conclusion, the contrast location and dispersion pattern, operator experience, and site and extent of nerve root compression have no influence on patients’ pain levels and global impression of change 4 weeks after CT-guided transforaminal epidural steroid injection in patients with single-level unilateral discogenic lumbar radiculopathy.

## Abbreviations

PGIC: Patient global impression of change
TFESI: Transforaminal epidural steroid injection
